# Hyperfunctioning of the right posterior superior temporal sulcus in response to neutral facial expressions presents an endophenotype of schizophrenia

**DOI:** 10.1038/s41386-020-0637-8

**Published:** 2020-02-14

**Authors:** Zhimin Yan, Stephanie N. L. Schmidt, Josef Frank, Stephanie H. Witt, Joachim Hass, Peter Kirsch, Daniela Mier

**Affiliations:** 1 Department of Clinical Psychology, Central Institute of Mental Health, University of Heidelberg/Medical Faculty Mannheim, Mannheim, Germany; 2 Department of Psychology, University Konstanz, Konstanz, Germany; 3 Department of Genetic Epidemiology in Psychiatry, Central Institute of Mental Health, University of Heidelberg/Medical Faculty Mannheim, Mannheim, Germany; 4 Department of Theoretical Neuroscience, Central Institute of Mental Health, University of Heidelberg/Medical Faculty Mannheim, Mannheim, Germany; 5 Faculty of Applied Psychology, SRH University of Applied Sciences Heidelberg, Heidelberg, Germany

**Keywords:** Schizophrenia, Personality

## Abstract

Deficits in social cognition have been proposed as a marker of schizophrenia. Growing evidence suggests especially hyperfunctioning of the right posterior superior temporal sulcus (pSTS) in response to neutral social stimuli reflecting the neural correlates of social-cognitive impairments in schizophrenia. We characterized healthy participants according to schizotypy (*n* = 74) and the single-nucleotide polymorphism *rs1344706* in ZNF804A (*n* = 73), as they represent risk variants for schizophrenia from the perspectives of personality traits and genetics, respectively. A social-cognitive fMRI task was applied to investigate the association of right pSTS hyperfunctioning in response to neutral face stimuli with schizotypy and *rs1344706*. Higher right pSTS activation in response to neutral facial expressions was found in individuals with increased positive (trend) and disorganization symptoms, as well as in carriers of the risk allele of *rs1344706*. In addition, a positive association between right–left pSTS connectivity and disorganization symptoms during neutral face processing was revealed. Although these findings warrant replication, we suggest that right pSTS hyperfunctioning in response to neutral facial expressions presents an endophenotype of schizophrenia. We assume that right pSTS hyperfunctioning is a vulnerability to perceive neutral social stimuli as emotionally or intentionally salient, probably contributing to the emergence of symptoms of schizophrenia.

## Introduction

Social-cognitive impairments have been proposed to present a marker of schizophrenia [1–4]. The impairments occur in different domains of social cognition, ranging from deficits in neutral face processing [5, 6], emotion recognition [7], up to complex social-cognitive processes [8], like inferring others’ mental states, known as theory of mind (ToM) [9], and are highly important for social functioning [10]. The association of these deficits to enhanced activity and connectivity of the right posterior superior temporal sulcus (pSTS [6, 11]) makes aberrant pSTS functioning during social cognition a highly promising endophenotype candidate for schizophrenia.

Several regions of the brain are central to social-cognitive processing, including amygdala, medial and inferior frontal cortex, insula, fusiform gyrus, as well as pSTS [12, 13]. Most of these regions have been both found to be affected structurally [14], as well as functionally [15, 16] in schizophrenia. For investigating the neural correlates of social-cognitive impairments in schizophrenia, we [17] developed a social-cognitive task that assesses several aspects of social cognition (namely neutral face processing (NFP), emotion recognition (ER), and affective ToM (aToM)) using facial expressions as stimuli. Applying this task, we found activation in key regions of social-cognitive processing in healthy participants [17]. In addition, hyperactivity in the right pSTS during NFP, but not during aToM, was revealed in two independent samples of patients with schizophrenia [6, 11]. Furthermore, we found hypoconnectivity between the right and left pSTS for aToM, and a relative hyperconnectivity between the right and left pSTS for NFP [6]. Other authors also [18, 19] showed hyperconnectivity of the pSTS in emotionally and intentionally neutral conditions of social-cognitive paradigms. Since the pSTS is a core area of social cognition and prominently involved in inferring other’s intentions [20] (also Schmidt et al., unpublished data), increased pSTS activation during NFP might be interpreted as a vulnerability for false-positive perceptions of intentions, also called hypermentalizing [21].

Imaging genetics studies with healthy participants and with relatives of patients with schizophrenia provided further evidence for aberrant pSTS functioning during social cognition as an endophenotype of schizophrenia. ZNF804A, a zinc-finger protein, presents an odds ratio of 1.08 (0.92–1.26 95% CI) for schizophrenia samples [22]. One of its single-nucleotide polymorphisms (SNPs, *rs1344706*) [23, 24] was identified in whole-genome association studies as the first common genetic variant associated with schizophrenia [25, 26]. *Rs1344706* is involved in regulating gene expression [27], and has been linked to executive functioning [28] and social cognition [29]. Imaging genetics findings from two healthy samples suggest that activity and connectivity of the STS and adjacent temporoparietal junction are associated with variation in *rs1344706* in a mentalizing task with sketches [29, 30]. Further, healthy relatives of patients with schizophrenia showed aberrant activation in this task. Family members had reduced activation in the medial prefrontal cortex during mentalizing, but increased activation in the posterior cingulate cortex and right middle temporal gyrus. Interestingly, activation in right middle temporal gyrus during mentalizing correlated positively with self-reported paranoid ideation [31]. These findings are exemplary of the approach to identify endophenotypes by investigating variations of traits of a disease in healthy participants.

Schizotypy as part of the schizophrenia spectrum is a valuable construct that refers to personality structures spreading dimensionally throughout the population [32, 33], but can also present as a personality disorder [34]. Schizotypy can be characterized by a three-factor model of sub-threshold psychotic symptoms, including positive (e.g., ideas of reference), negative (e.g., no close friends), and disorganization symptoms (e.g., eccentric behavior). Several studies have consistently revealed an association between schizotypy and the development of a psychotic disorder (for a review please see ref. [35]). A longitudinal study reported that 9% of an at-risk sample had a transition to schizophrenia within 12 months, and suggested self-reported schizotypy presenting the most reliable scale-based predictor [36]. In addition, accumulating evidence points to schizotypy and schizophrenia having common genetic [37], neuroanatomical [38], and neurocognitive [39] abnormalities, which again highlights the strong associations between schizotypy and schizophrenia. Importantly, as in schizophrenia, schizotypy has been associated with different kinds of social-cognitive deficits [40, 41].

To date, only two fMRI studies have investigated the association between neural correlates of mentalizing and schizotypy in healthy participants. Both studies found right pSTS activity for mentalizing varying with schizotypy [40, 42]. However, whereas one [42] revealed negative symptoms to be positively related to right pSTS activation during mentalizing, the other [40] showed a positive association with positive symptoms. In these studies, different tasks and accordingly different stimulus materials were used to investigate ToM, possibly explaining the divergent results. An even more general and crucial aspect when comparing social-cognitive studies, however, is not only the selection of stimulus material but also of the control condition (ranging from emotionally neutral analogs of the experimental condition to completely nonsocial conditions), which is usually subtracted from the higher-order social-cognitive process. Therefore, divergent findings in prior studies might be explained by differences in brain activation in the control condition between participants with and without schizophrenia risk.

To summarize, deficits in social cognition are proposed to present a marker for schizophrenia [1], and aberrant pSTS functioning during social cognition is a promising endophenotype of schizophrenia [29, 30]. In this imaging genetics study, we applied a social-cognitive fMRI task [6, 11] that assesses different social-cognitive processes, and has consistently revealed right pSTS hyperactivation during NFP, but not during mentalizing, in patients with schizophrenia [6, 11]. We aimed at investigating whether we find in healthy participants a comparable activation and connectivity pattern as in schizophrenia, depending on the *ZNF804A rs1344706* risk allele, and schizotypy. This allowed us to assess possible pSTS hyperfunctioning unconfounded of medication status, or chronicity of disease. For both *ZNF804A rs1344706* risk allele and schizotypy, previous studies found a relationship to aberrant pSTS activation during mentalizing [29, 30, 40, 42], but the response to neutral facial expressions was not investigated. We hypothesized that activation and connectivity of the right pSTS in response to neutral facial expressions in healthy participants is positively associated with (1) the risk allele of the *rs1344706* genotype and (2) higher self-reported schizotypy.

## Materials and methods

### Participants

Of 81 healthy participants, seven were excluded for the present analyses: five due to low fMRI data quality, one due to a BDI-II [43] score >18, and one due to schizotypy scores >3 SD above the group mean. For the genetics analyses, one additional participant was excluded because genotyping for rs1344706 was not possible. Therefore, we included 74 participants (40 females, see Table 1) in the behavioral and imaging analyses and 73 participants (39 females) for the imaging genetic analyses. Participants were grouped for the imaging genetics analysis for the existence of the risk allele of schizophrenia [25]; ZNF804A genotype groups: 62 AA/CA (risk-allele carriers and 11 CC non-risk-allele carriers). All participants were of German ancestry, had higher school certificate, were right-handed, had normal or corrected-to-normal vision and no self-reported background of mental or neurological disorders, or drug abuse. In addition, participants reported having no relatives with psychotic disorders.

**Table 1:** Characteristics of the sample.

	Whole sample (*n* = 74)		Range of SPQ scores (*n* = 74)		AA/CA (*n* = 62)		CC (*n* = 11)		Genotype effect	
	Mean	SD	Min	Max	Mean	*SD*	Mean	*SD*	*t*	*p*
Age	23.50	3.83	–	–	23.27	3.80	24.36	3.98	−0.87	0.387
SPQ total	11.00	9.09	0	37	11.54	9.08	7.82	9.28	1.25	0.215
Positive symptoms	5.05	4.86	0	21	5.18	4.93	4.09	4.68	0.68	0.500
Negative symptoms	3.05	3.17	0	13	3.29	3.31	1.82	2.14	1.42	0.226
Disorganization symptoms	2.89	2.92	0	12	3.08	2.93	1.90	2.91	1.22	0.215

*SPQ* schizotypal personality questionnaire, *AA/CA* indicates the risk-allele carriers, *CC* indicates the non-risk-allele carriers.

Prior to the study, participants were informed about study procedure and purpose and gave their written informed consent. The study was approved by the local ethics board of the Medical Faculty Mannheim, University of Heidelberg. The data reported here are part of a larger study on the human mirror neuron system that involved a measurement containing simultaneous EEG-fMRI with three tasks (including an imitation task, an empathy task, and the social-cognitive task presented here), blood-taking and a series of questionnaires, including the Schizotypal Personality Questionnaire (SPQ [44], details are presented in the Supplementary Text 1), and a second measurement with transcranial magnetic stimulation prior to fMRI. The results reported in this paper are based on the fMRI data of the first appointment.

### Experimental paradigm

**Fig. 1:**
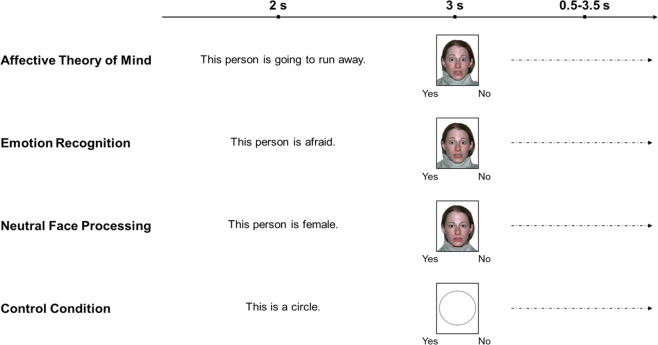
The social cognition fMRI task.

We applied a modified version of the social-cognitive task that was used in earlier studies with patients with schizophrenia [6, 11]. The paradigm was extended to four conditions, including three levels of social cognition [lower-level social cognition (NFP), ER, and higher-level social cognition (aToM)], and a nonsocial control condition. In each trial of the social-cognitive conditions, a statement preceded a facial expression. These statements described the facial expressions referring to physical features (gender or age) for NFP, the emotional state (fear or anger) for ER, or the possible intention (running away or blustering) for aToM. For NFP, only neutral facial stimuli were shown, for ER and aToM only emotional facial expressions. The facial stimuli were taken from the Karolinska Directed Emotional Faces set [45]. Half of the stimulus persons were male, and the same persons were used for each of the social-cognitive conditions. For the control condition, prior to a geometric figure (a triangle or a circle) a statement describing the figure (e.g., “This is a circle”) was displayed. Task duration was around 8 min (details of timing and presentation can be found in Fig. 1 and Supplementary Text 2).

### Genotyping

Genotypes for *ZNF804A SNP rs1344706* were extracted from whole-genome genotype data obtained using Illumina Global Screening array and following stringent quality control (see Supplementary Text 3).

### Imaging data acquisition and analyses

The study was conducted with a 3-Tesla Siemens Tim TRIO whole-body magnetic resonance tomography (Siemens Medical Systems, Erlangen, Germany; acquisition protocol can be found in Supplementary Text 4). Brain activity and connectivity analyses were conducted with SPM8 (Wellcome Department of Imaging Neuroscience, Institute of Neurology, London, UK). Data preprocessing contained slice-time correction, realignment, co-registration to the structural image, spatial normalization (MNI template) with resampling to a 3 × 3 × 3 mm voxel size, and spatially smoothing with an 8 mm full-width half-maximum kernel. The first-level analyses were achieved by a general linear model with four regressors (aToM, ER, NFP, and control), and six motion parameters, derived from the realignment procedure, as covariates. The hemodynamic response function was modeled to the onset times of the pictures. The time series was high-pass filtered using a 128 Hz function. From the model, linear combinations of the regressors built the contrasts of interest, including effects of each higher against the lower social-cognitive condition (aToM > ER, ER > NFP), and each condition against control (aToM >control, ER > control, NFP > control). Connectivity analyses were applied using generalized psychophysiological interactions (gPPI [46]), as implemented in the gPPI toolbox (http://www.nitrc.org/projects/gppi) with a functional mask of right STS as seed region, produced from our previous comparison between aToM and ER in 40 undergraduate students [17] (details of the gPPI analysis are reported in Supplementary Text 4).

For second-level analyses, significance threshold for exploratory whole-brain analyses was *p* < 0.05 FWE-corrected, *k* = 10. We conducted one sample *t* tests to analyze the effect of each social-cognitive condition, and a within-subject one-way analysis of variance (ANOVA) to identify the neural correlates of increased social-cognitive processing (contrast: [aToM > control] > [ER > control] > [NFP > control]). Regression analyses were conducted to explore the associations between the factors related to schizophrenia (schizotypy and the rs1344706 risk allele) and right pSTS activation, and connectivity for the different social-cognitive conditions. Region of interests were right and left pSTS, as derived from an earlier study [17]. These masks were also applied in our studies on patients with schizophrenia [6, 11]. The significance threshold for the ROI analyses was set to *p*_*FWE*_ < 0.05 small volume corrected (svc), *k* = 10. Behavioral data were analyzed with SPSS version 23. Differences between the social-cognitive conditions in reaction times (RTs) or accuracy were analyzed with repeated measures ANOVA, post hoc tests were conducted with paired-samples *t* tests. Pearson correlation was applied to investigate possible associations among task conditions, and to test the associations between schizotypy and task performance. We conducted independent sample *t* tests to test genotype effects on task performance, as well as to investigate differences in self-reported schizotypy, depending on genotype.

## Results

### Behavior

Similar to our previous studies [6, 11, 17], RTs and accuracy differed significantly between conditions, with the control condition being the easiest and aToM being the most difficult task condition. Neither genotype nor schizotypy significantly affected task performance (detailed behavioral results are reported in Supplementary Text 5 and Supplementary Fig. 1). In addition, no significant differences in self-reported schizotypy were revealed, depending on the risk allele (see Table 1).

### Imaging

**Fig. 2:**
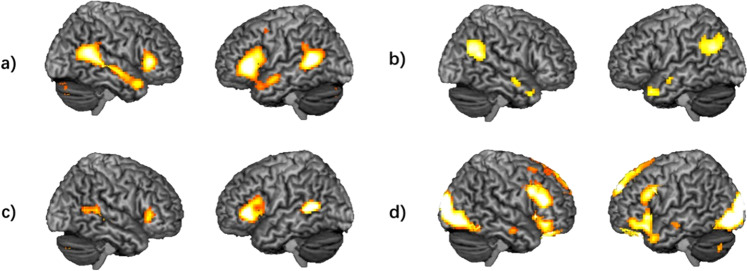
Neural correlates of distinct social-cognitive processes. **a)** neural correlates of increasing social-cognitive demands [with the contrast: (affective theory of mind > control) > (emotion recognition > control) > (neutral face processing > control)]; **b)** affective theory of mind (> emotion recognition); **c)** emotion recognition (> neutral face processing); **d)** neutral face processing (> control condition). Significance threshold is *p* < 0.05, FWE-corrected, *k* = 10.

Replicating the results from our previous studies [6, 17], activation increased linearly from NFP over ER to aToM in regions of the “social brain”, including bilateral superior temporal gyrus covering pSTS, bilateral inferior frontal gyrus covering BA44 (Fig. 2; detailed results of task effects are presented in Supplementary Table 1). Whole-brain analyses of right pSTS connectivity differences between conditions were not significant across participants. ROI analyses revealed greater pSTS connectivity between hemispheres for aToM compared with ER at the trend level (peak voxel: −57, −49, 7; *t* = 3.37, *p*_*FWE*_ = 0.069 svc, *k* = 10).

#### rs1344706

**Fig. 3:**
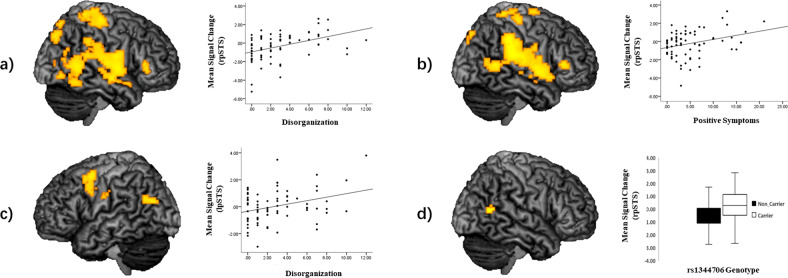
Associations between functioning of right posterior superior temporal sulcus for neutral face processing and schizotypy as well as rs1344706 genotype. The first two scatter plots show positive correlations between activation in the right posterior superior temporal sulcus (pSTS) for neutral face processing (> control) and **a)** disorganization, as well as **b)** positive symptoms; **c)** positive association of disorganization with right-to-left pSTS connectivity for neutral face processing (> control); **d)** genotype effect of increased activation in the right pSTS for neutral face processing (> control). Significance threshold for display purposes is *p* < 0.005 uncorrected, *k* = 10. Note: rpSTS stands for right posterior superior temporal sulcus, lpSTS stands for left posterior superior temporal sulcus.

ROI analyses revealed that risk-allele carriers compared with non-risk-allele carriers had increased right pSTS activation during NFP (> control; peak voxel: 63, −58, 13; *t* = 3.19, *p*_*FWE*_ = 0.042 svc, *k* = 10, Fig. 3). No significant differences in pSTS activation were found for ER and aToM, and also right–left pSTS connectivity during all task conditions did not differ between risk-allele carriers and non-risk-allele carriers. In addition, whole-brain analyses with the given significance threshold revealed no significant group differences, neither in the activation nor in the connectivity analyses.

#### Schizotypy

There was a trend for a positive association between right pSTS activation for NFP (> control) and schizotypy sum score (peak voxel: 63, −55, 10; *t* = 3.01, *p*_*FWE*_ = 0.065 svc, *k* = 10). There was also a significant positive association between activation in right pSTS and disorganization symptoms (peak voxel: 57, −55, 7; *t* = 3.54, *p*_*FWE*_ = 0.018 svc, *k* = 10, Fig. 3), and at the trend level with positive symptoms (peak voxel: 63, −55, 10; *t* = 3.94, *p*_*FWE*_ = 0.077 svc, *k* = 10, Fig. 3). ROI analysis also revealed a significant positive correlation between disorganization symptoms and right–left pSTS connectivity during NFP (> control; peak voxel: −45, −70, 22; *t* = 3.60, *p*_*FWE*_ = 0.038 svc, *k* = 10, see Fig. 3). Neither for ER nor for aToM were significant associations between schizotypy and pSTS activation, or connectivity found. In addition, whole-brain analyses with the given significance threshold did not reveal any significant associations of schizotypy with task-related brain activation and connectivity.

## Discussion

This study aimed at investigating whether pSTS functioning during social-cognitive processing is an endophenotype for schizophrenia. Confirming our hypothesis, we found a positive association of right pSTS activation for neutral face processing with schizotypy (in particular disorganization, and positive symptoms on a trend level), and also with a risk allele for schizophrenia. Furthermore, connectivity between the right and left pSTS during neutral face processing was positively associated with disorganization symptoms.

The pSTS is consistently found to be involved in inferring goals and intentions [6, 11, 17, 20]. Across participants, we replicated our previous findings showing decreased performance and increased activation in pSTS and BA44 with increasing social-cognitive demands. With this, our results again highlighted the role of pSTS functioning for inferring others’ intentions [20]. Importantly, increased right pSTS functioning in our participants with schizophrenia risk allele and subclinical symptoms of schizophrenia was present only for neutral face processing, but not for higher-order social-cognitive conditions. This supports our previous findings and conclusions that impairments in higher-order social cognition in schizophrenia might originate in impaired basic social-cognitive processes [6, 11]. Our results are also consistent with further previous findings with patients with schizophrenia. A recent study reported not only increased pSTS activation in response to the emotionally and intentionally neutral control condition in their social-cognitive task but also increased pSTS connectivity [18]. These results add to the idea that pSTS dysfunction for neutral social stimuli might be regarded as neural basis for hypermentalizing, which may constitute a vulnerability to the emergence of delusion [6].

But how could this pSTS hyperfunctioning in response to neutral facial expressions cause symptoms of schizophrenia? Kapur [47, 48] proposed that psychosis, particularly delusions, result from aberrant attribution of novelty and salience to objects and associations, and that faulty attributions of salience arise due to chaotic, context-inappropriate firing of dopamine neurons. Delusions have been suggested to represent a deficit in encoding the precision of predictions and prediction errors [49], indicating a bottom-up inappropriate perceptual input; i.e., aberrant salience. Supporting this idea, our results revealed a positive association between self-reported symptoms of schizotypy, as well as between the presence of a risk allele for schizophrenia, and activation in the right pSTS for neutral faces, and unveiled a positive association between disorganization and right-to-left pSTS connectivity. This might indicate that people with higher positive symptoms and a genetic risk for schizophrenia might be prone to perceive neutral faces as emotionally or intentionally salient. Whether these inappropriate perceptual inputs lead to delusions in turn could depend on how individuals interpret these false perceptions, pointing to the importance of a top-down cognitive explanation to delusions [47, 48], and the impact of disorganization. When the cognitive explanation is interfered or interrupted due to executive dysfunction, the accumulating experiences of aberrant salience might gradually increase confusion and result in delusional ideas that are based on overinterpretation of emotions and intentions to neutral social stimuli.

Together, our findings add to the perspective that delusions probably derive from dynamic interactions between bottom-up erroneous perception and top-down cognitive deficits, caused by increased responsiveness to emotionally and intentionally neutral social stimuli [21]. Since all of our participants were without a history of mental disorders, we found alterations only on the level of neural functioning. Further studies with large patient samples that allow the analysis of subgroups are needed to investigate the validity of the proposed mechanisms in schizophrenia.

Importantly, aberrantly high pSTS functioning in response to neutral social stimuli seems to be not “only” a marker of schizophrenia, but an endophenotype of schizophrenia according to the criteria characterizing an endophenotype proposed by Gottesman and Gould [50]: (1) the endophenotype is associated with illness in the population: aberrant right pSTS functioning is consistently observed in patients with schizophrenia in response to stimuli and situations without emotional, or intentional meaning [5, 6, 11, 18, 19, 51]. Our current results show a comparable neural pattern in healthy participants with increased proneness to schizophrenia, illustrating an association of right pSTS dysfunction with schizophrenia symptoms also in healthy participants. (2) The endophenotype is heritable: in line with previous studies showing aberrant pSTS functioning in schizophrenia risk-allele carriers [29, 30], we found increased right pSTS activity in response to the neutral condition in *rs1344706* risk-allele carriers, possibly reflecting one aspect of the heredity of the right pSTS dysfunction. (3) The endophenotype should be state-independent: we found the neural pattern first in schizophrenia out-patients who were remitted from positive pathology [11], then in in-patients with schizophrenia [6], now even in healthy participants carrying the psychosis allele, suggesting that right pSTS dysfunction might represent a state-independent neural pattern for schizophrenia. (4) Within families, endophenotype and illness co-segregate: increased engagement of right pSTS varied with positive symptoms in patients with schizophrenia’ relatives [31], suggesting that right pSTS dysfunction and schizophrenia symptoms co-segregate within families. However, studies systematically investigating differences in pSTS functioning between relatives of patients with schizophrenia are pending. (5) The endophenotype in affected family members is found at a higher rate in non-affected family members than in healthy participants: While hyperfunctioning was observed in relatives of patients with schizophrenia who reported positive symptoms, it is also found in non-affected family members at a higher rate than in healthy participants without familial risk for schizophrenia [31]. In addition to the criteria initially proposed by Gottesman and Gould, a further criterion has been put forward [52]: (6) the endophenotype should be a trait that can be measured reliably, and ideally is more strongly related with the disease of interest than with other psychiatric conditions: aberrant activation in the right pSTS was consistently revealed by our social-cognitive task in patients with schizophrenia [6, 11] and also in the current study in healthy participants with increased schizophrenic proneness, but not in patients with borderline personality disorder [53]. In addition, especially in patients with schizophrenia with paranoid symptoms, pSTS activity during a neutral condition was higher than in patients with autism [18, 54], highlighting that dysfunction in right pSTS is not only a reliably assessable trait but might be specific to schizophrenia. Thus, there is extensive evidence supporting the idea that hyperfunctioning of pSTS to neutral social stimuli represents an endophenotype for schizophrenia.

Despite the reported studies consistently finding genotype effects on brain activation and connectivity [29, 30], they, like this study, tested only one risk SNP’s effect. In addition, since genetic penetrance is higher for endophenotypes than phenotypes [55]; i.e., significant association between the risk allele and right pSTS functioning, but no significant association between the risk allele and schizotypy, several approaches would be of interest for future studies to validate our findings and to investigate the proposed mechanisms: (a) investigating the load of risk SNPs to reveal biological subcategories of schizophrenia [56], (b) due to unequal distribution of risk-allele presence (only 11 participants homozygous for the non-risk allele), replication of the finding with pre-selection of participants depending on their genotype, (c) replicating the marginally significant association of positive symptoms and right pSTS activation with different approaches to assess schizotypy, such as the Oxford-Liverpool Inventory of Feelings and Experiences [57], (d) targeting not only right pSTS activation and connectivity, but also of further regions that are central for social-cognitive processing (e.g., amygdala, MPFC). Besides, some previous studies only reported hypo-functioning in the pSTS with regard to schizophrenia in response to higher-level social cognition (such as ToM) [29, 30]. Whether these studies would also reveal pSTS hyperfunctioning if the neutral condition was investigated remains an open question. However, investigating pSTS hyperactivation in participants with schizophrenia risk can be challenging, because no significant pSTS activation in response to the neutral conditions is found across all participants, pointing to small effect sizes that warrant moderate to large sample sizes to reveal the effects. Moreover, some studies suggest aberrations in left pSTS instead of the right pSTS presenting an intermediate phenotype for schizophrenia [29, 30]. Perner et al. [58] proposed that the left pSTS is linked to perspective differences for mental and nonmental objects, while the right pSTS is associated with mental states. Future studies should approach the question of laterality with a systematic variation of social-cognitive processing to clarify the functioning of this region in schizophrenia.

Taken together, our findings point to right pSTS hyperfunctioning in response to neutral faces as an endophenotype of schizophrenia. We assume that right pSTS hyperfunctioning presents a vulnerability to perceive neutral social stimuli as emotionally or intentionally salient and suggest that bottom-up and top-down aberrations interact to cause delusions via deficient social perception.

## Funding and disclosure

This work was supported by the Heidelberg Academy of Science and Humanities. Zhimin Yan is supported by the Chinese Scholarship Council. The authors declare no conflict of interest. Open access funding provided by Projekt DEAL.

## Supplementary information

Supplement
