# Supplementary material for: Hyperfunctioning of the right posterior superior temporal sulcus in response to neutral facial expressions presents an endophenotype of schizophrenia
Source: Neuropsychopharmacology. 2020 Feb 14;45(8):1346–52. doi: 10.1038/s41386-020-0637-8 (PMC7297989; doi:10.1038/s41386-020-0637-8)
Supplement: Supplementary file 1 — Supplement [file 41386_2020_637_MOESM1_ESM.doc]

**Supplement:**

**Hyperfunctioning of right posterior superior temporal sulcus in response to neutral facial expressions presents an endophenotype of schizophrenia**

*** MSc Zhimin Yan^1^, * MSc Stephanie N.L. Schmidt^1,2^, MSc Josef Frank^3^, PhD Stephanie H. Witt^3^, Prof. Joachim Hass^4,5^, Prof. Peter Kirsch^1^, Prof. Daniela Mier+^1,2^**

^1^ Department of Clinical Psychology, Central Institute of Mental Health, University of Heidelberg / Medical Faculty Mannheim, Mannheim, Germany

^2^ Department of Psychology, University Konstanz, Konstanz, Germany

^3^ Department of Genetic Epidemiology in Psychiatry, Central Institute of Mental Health, University of Heidelberg / Medical Faculty Mannheim, Mannheim, Germany

^4^ Department of Theoretical Neuroscience, Central Institute of Mental Health, University of Heidelberg / Medical Faculty Mannheim, Mannheim, Germany

^5^ Faculty of Applied Psychology, SRH University of Applied Sciences Heidelberg, Heidelberg, Germany

*both authors contributed equally

+Corresponding author:

Daniela Mier

Box 905

Universitaetsstrasse 10

78457 Konstanz

Email: [Daniela.Mier@uni-konstanz.de](mailto:Daniela.Mier@uni-konstanz.de)

Phone: +49 7531-884762

Fax: +49 7531-882891

**Content**

[Supplementary Texts 3](#__RefHeading___Toc534222795)

[Supplementary Text 1: Questionnaire 3](#__RefHeading___Toc534222796)

[Supplementary Text 2: Details of timing and presentation of the social-cognitive task 4](#__RefHeading___Toc534222797)

[Supplementary Text 3: Genotyping 5](#__RefHeading___Toc534222798)

[Supplementary Text 4: Functional imaging data acquisition and analyses 6](#__RefHeading___Toc534222799)

[Supplementary Text 5: Behavioral results 7](#__RefHeading___Toc534222800)

[Supplementary Figure 8](#__RefHeading___Toc534222801)

[Supplementary Table 9](#__RefHeading___Toc534222802)

# Supplementary Texts

## Supplementary Text 1: Questionnaire

The Schizotypal personality questionnaire (SPQ) includes 74 items with a dichotomous response format [Yes(1)/No(0)] and consists of nine subscales which represent the DSM-IV criteria for schizotypal personality disorders. The subscales can be grouped by three factors: positive (magical ideation, paranoid ideation, perceptual aberrations, ideas of reference), negative (constricted affect, no close friends, social anxiety), and disorganization (odd speech, eccentric behavior) symptoms. Cronbach’s alpha for the whole SPQ in the present study is 0.91, for the subscale of positive symptoms 0.86, for the subscale of the negative symptoms 0.82, for the subscale of disorganization 0.79.

## Supplementary Text 2: Details of timing and presentation of the social-cognitive task

Each trial started with a statement for 2 s, followed by a picture (a facial expression or a geometric figure) with the choice “yes” or “no” underneath. Participants were asked to respond whether the preceding statement matches the current picture by pressing the corresponding button within 3 s. In 50% of trials, the statement matched the following picture. Trials were separated by a fixation cross of a mean duration of 2 s (with a jitter of 0.5–3.5 s). Each condition had 20 trials resulting in an experimental time of 8 min approximately. The task was implemented with Presentation software, version 9.50 (Neurobehavioral Systems Albany, CA, USA). Participants responded with a current design response device (Current Designs, Inc., Philadelphia, PA) and watched the experiment via VisuaStim video goggles (Resonance Technology Inc, Northridge, USA).

## Supplementary Text 3: Genotyping

DNA was extracted from full blood using PerkinElmer chemagen (Baesweiler, Germany) chemagic 360 DNA extraction system. Genotyping was performed using Illumina (San Diego, CA, USA) Global Screening Array bead chips. Resulting genotypes were subjected to stringent quality control (QC). This included removal of DNA samples with either insufficient quality (individual missing rate > 2%, based on prefiltered SNPs with call rate > 0.95), discrepancies between phenotypic and genotype based sex, or heterozogosity deviation (autosomal |F_ST_| > 0.2), and removal of SNPs with insufficient call rate (CR<0.98), deviation from Hardy-Weinberg equilibrium (p_HWE_ < 1x10^-6^), or low minor allele frequency (MAF < 0.01), as well as removal of genetic outliers. No individual failed QC tests.

For the present study genotypes of SNP rs1344706 were finally extracted from the quality-controlled data set described above.

## Supplementary Text 4: Functional imaging data acquisition and analyses

Prior to functional imaging, we acquired a T1-weighted anatomical scan (TR = 1570 ms, TE = 2.75 ms; flip angle = 15°, field of view = 256 mm; matrix = 256x256; voxel size 1x1x1 mm). Functional scans were obtained by conducting a T2*-weighted gradient echo planar imaging sequence (TR = 2000 ms; TE = 30 ms; flip angle 80 degree; field of view = 192 mm; matrix: 64 x 64 mm). Each volume consisted of 32 slices, acquired in a descending order with a slice-thickness of 3 mm with 1 mm gap (voxel size: 3 x 3 x 4 mm^3^).

For connectivity analyses of right pSTS connectivity, the first eigenvariate of the seed region was extracted for each person (no significance threshold was applied for eigenvariate extraction), deconvolved with the canonical hemodynamic response function (HRF) and multiplied with time series of each, affective ToM, emotion recognition, neutral face processing, and control conditions to represent condition-specific interactions. These interaction regressors were subsequently convolved again with the HRF.

## Supplementary Text 5: Behavioral results

A significant main effect of condition in RTs (F = 465.58, *p* < .001,
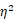
 = .86) and in accuracy (F = 44.32, *p* < .001,
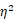
 = .39) was found (see Figure 2). Post-hoc tests revealed that the RTs in the control condition were significantly shorter than in the neutral face processing condition (*t* (73) = -7.00, *p* < .001, *d* = -.81), in the emotion recognition condition (*t* (73) = -27.84, *p* < .001, *d* = -3.24), as well as in the affective ToM condition (*t* (73) = -30.68, *p* < .001, *d* = -3.57); the RTs in the neutral face processing condition were shorter than in the emotion recognition condition (*t* (73) = -19.61, *p* < .001, *d* = -2.28) and in the affective ToM condition (*t* (73) = -20.79, *p* < .001, *d* = -2.42). No significant difference of RTs between the emotion recognition condition and the affective ToM condition was found (*t* (73) = .35, *p* = .73). In terms of accuracy, post-hoc tests showed participants presented better performance in the control condition than in the neutral face processing condition (*t* (73) = 11.34, *p* < .001, *d* = 1.32), in the emotion recognition condition (*t* (73) = 8.75, *p* < .001, *d* = 1.02), as well as in the affective ToM condition (*t* (73) = 10.73, *p* < .001, *d* = 1.25); and lower accuracy in the affective ToM condition was found in comparison to the neutral face processing condition (*t* (73) = -2.74, *p* = .008, *d* = -.32) and the emotion recognition condition (*t* (73) = -2.67, *p* = .009, *d* = -.31). No significant differences in the accuracy were revealed between neutral face processing and emotion recognition (*t* (73) = .63, *p* = .53). Furthermore, correlation analyses only showed a significant association of the accuracy in the affective ToM condition and in the emotion recognition condition (*r* = .42, *p* < .001, *d* = .94).

No significant performance differences between risk- and non-risk-alle carriers occurred. Also no significant correlations between schizotypy scores and performance were found.

# Supplementary Figure

Supplementary Figure 1. Behavioral results of the social-cognitive fMRI task. Mean of reaction times (left) and of accuracy (right) for the four experimental conditions.

**
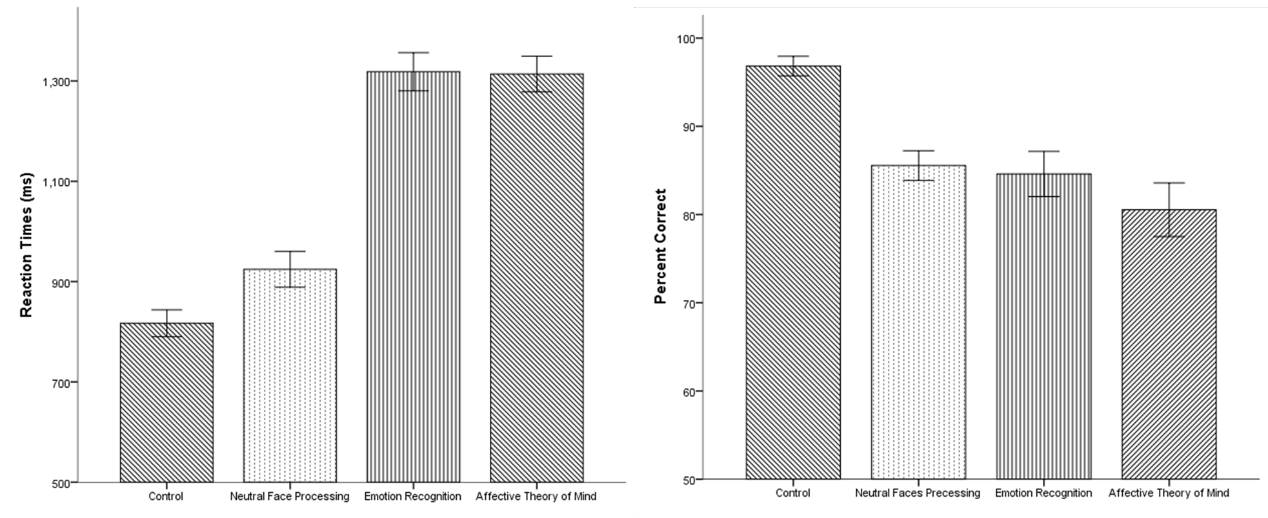
**

# Supplementary Table

| Supplementary Table 1. Brain activation in the social-cognitive task across all participants. | | | | | | | | |  |
| --- | --- | --- | --- | --- | --- | --- | --- | --- | --- |
| Area | L/R | Cluster | | MNI | | | t-value | p-value |  |
|  |  |  |  | x | y | z |  |  |  |
| **aToM > ER** | | | | | | | | |  |
| *Whole Brain Analyses* | | | | | | | | |  |
| Superior Temporal Gyrus | R | | 330 | 63 | -52 | 19 | 7.60 | < 0.001 |  |
| Superior Temporal Gyrus | L | | 330 | -45 | -61 | 28 | 7.23 | < 0.001 |  |
| Superior Temporal Gyrus | L | | 58 | -45 | 17 | -29 | 6.77 | < 0.001 |  |
| Middle Temporal Gyrus | R | | 15 | 57 | -7 | -14 | 5.80 | < 0.001 |  |
| Superior Temporal Gyrus | R | | 11 | 45 | 14 | -32 | 5.64 | < 0.001 |  |
| Middle Temporal Gyrus | L | | 11 | -60 | -7 | -11 | 5.42 | < 0.001 |  |
| *Region of Interest Analyses* | | | | | | | | |  |
| Superior Temporal Sulcus | L | |  | -45 | -58 | 22 | 5.99 | < 0.001 |  |
| Superior Temporal Sulcus | R | |  | 63 | -52 | 19 | 7.60 | < 0.001 |  |
|  |  | |  |  |  |  |  |  |  |
| **ER > NF** | | | | | | | | |  |
| *Whole Brain Analyses* | | | | | | | | |  |
| Inferior Frontal Gyrus | L | | 379 | -51 | 32 | 4 | 10.22 | < 0.001 |  |
| Superior Temporal Gyrus | L | | 224 | -51 | -52 | 10 | 9.61 | < 0.001 |  |
| Superior Temporal Gyrus | R | | 165 | 45 | -40 | 10 | 6.73 | < 0.001 |  |
| Inferior Frontal Gyrus | R | | 79 | 48 | 26 | -2 | 6.65 | < 0.001 |  |
| Cingulate Gyrus | L | | 21 | -6 | 20 | 46 | 5.70 | 0.004 |  |
| Cerebellum | R | | 13 | 27 | -76 | -38 | 5.75 | 0.004 |  |
| *Region of Interest Analyses* | | | | | | | | |  |
| Superior Temporal Sulcus | L | |  | -54 | -52 | 10 | 9.33 | < 0.001 |  |
| Superior Temporal Sulcus | R | |  | 51 | -52 | 10 | 6.00 | < 0.001 |  |
|  | | | | | | | | |  |
| **NF > Control** | | | | | | | | |  |
| *Whole Brain Analyses* | | | | | | | | |  |
| Occipital Lobe | R | | 2736 | 12 | -97 | 13 | 18.39 | < 0.001 |  |
| Frontal Lobe | L | | 969 | -6 | 59 | 34 | 10.32 | < 0.001 |  |
| Inferior Frontal Gyrus | R | | 536 | 45 | 32 | 16 | 11.92 | < 0.001 |  |
| Inferior Frontal Gyrus | L | | 444 | -39 | 23 | -17 | 9.06 | < 0.001 |  |
| Rectal Gyrus | R | | 381 | 3 | 38 | -20 | 11.50 | < 0.001 |  |
| Inferior Frontal Gyrus | R | | 324 | 36 | 32 | -11 | 12.19 | < 0.001 |  |
| Middle Frontal Gyrus | L | | 216 | -42 | 11 | 31 | 9.21 | < 0.001 |  |
| Cingulate Gyrus | L | | 41 | 0 | -52 | 28 | 6.69 | < 0.001 |  |
| Parahippocampal Gyrus | R | | 37 | 30 | -7 | -32 | 8.14 | < 0.001 |  |
| Parahippocampal Gyrus | R | | 36 | 21 | -10 | -14 | 8.68 | < 0.001 |  |
| Middle Frontal Gyrus | R | | 30 | 36 | 14 | 61 | 6.09 | 0.001 |  |
| Cerebellum | L | | 20 | 0 | -55 | -32 | 7.21 | < 0.001 |  |
| Inferior Temporal Gyrus | R | | 18 | 63 | -10 | -20 | 6.57 | < 0.001 |  |
| Parahippocampal Gyrus | L | | 14 | -18 | -10 | -14 | 6.30 | 0.001 |  |
| Middle Temporal Gyrus | L | | 12 | -63 | -16 | -14 | 5.71 | 0.005 |  |
| Cerebellum | L | | 11 | -9 | -79 | -32 | 6.42 | < 0.001 |  |
| Cerebellum | L | | 10 | -33 | -70 | -44 | 6.45 | < 0.001 |  |
| *Region of Interest Analyses* | | | | | | | | |  |
| Superior Temporal Sulcus | R | |  | 51 | -61 | 22 | 4.91 | < 0.001 |  |
|  | | | | | | | | |  |
| **(aToM > control) > (ER > control) > (NF>control)** | | | | | | | | |  |
| *Whole Brain Analyses* | | | | | | | | |  |
| Superior Temporal Gyrus | R | | 942 | 48 | -37 | 4 | 10.88 | < 0.001 |  |
| Inferior Frontal Gyrus | L | | 712 | -51 | 32 | 4 | 12.2 | < 0.001 |  |
| Superior Temporal Gyrus | L | | 591 | -57 | -52 | 10 | 11.03 | < 0.001 |  |
| Inferior Frontal Gyrus | R | | 192 | 54 | 29 | 4 | 9.86 | < 0.001 |  |
| Cerebellum | R | | 171 | 27 | -73 | -35 | 6.96 | < 0.001 |  |
| Cerebellum | L | | 72 | -21 | -79 | -32 | 7.54 | < 0.001 |  |
| Superior Frontal Gyrus | L | | 60 | -3 | 11 | 61 | 5.8 | < 0.001 |  |
| Precuneus | L | | 27 | -6 | -67 | 40 | 6.26 | < 0.001 |  |
| Cerebellum | L | | 25 | -9 | -40 | 22 | 5.9 | < 0.001 |  |
| Middle Frontal Gyrus | L | | 15 | -39 | 5 | 43 | 5.34 | 0.004 |  |
| Cerebellum | R | | 12 | 21 | -10 | 31 | 5.03 | 0.013 |  |
| *Region of Interest Analyses* | | | | | | | | |  |
| Superior Temporal Sulcus | L | |  | -57 | -52 | 10 | 11.03 | < 0.001 |  |
| Superior Temporal Sulcus | R | |  | 60 | -52 | 16 | 9.64 | < 0.001 |  |
| Note: 1. aToM = affective Theory of Mind, ER = emotion recognition, NF = neutral face processing. 2. Significance threshold is p < 0.05 FWE-corrected, k = 10. | | | | | | | | |  |
